# On building cytomorphology skills: Influence of slide reviewing habits on diagnostic competency in cytology

**DOI:** 10.1111/cyt.13150

**Published:** 2022-06-09

**Authors:** Paul Z. Chiou, Yuane Jia

**Affiliations:** ^1^ Department of Clinical Laboratory and Medical Imaging Sciences, School of Health Professions Rutgers University Newark New Jersey USA; ^2^ Department of Interdisciplinary Studies, School of Health Professions Rutgers University Newark New Jersey USA

**Keywords:** chronobiology, cytology, diagnostic competency, gynecological cytology, laboratory education, pathology

## Abstract

**Objectives:**

The specific aims of the study are to assess whether the amount of time a cytology learner spends reviewing slides correlates with increased diagnostic competency and to determine whether time spent reviewing slides immediately after the multi‐head sessions correlates with a higher level of proficiency. The paper also seeks to explore the impacts of the time of day at which slides were reviewed on diagnostic learning outcome.

**Methods:**

Data obtained through the cytology laboratory screening logs were reviewed, and the number of hours per day and the times of the day at which the students were present at the glass slide library were tabulated and compared against each of the seven‐unit slide exam scores in the semester to explore possible relationships.

**Results:**

There was a positive linear relationship (*r* = 0.29) between the number of hours students spent in the laboratory reviewing cases and competency. When the students' unit diagnostic test scores were classified into low and high categories for each test, there was a significant correlation (P = 0.008) between a lack of time spent screening slides in the lab and the number of times a student was ranked at the bottom of the class. Our data do not support a recency effect nor a difference in test scores between those who reviewed cases in the morning vs in the afternoon.

**Conclusions:**

While educating and training a strong cytology workforce may be challenging, our study provides new insights and sheds light on the importance of spending time reviewing slides, and provides guidance for struggling students on how best to improve. Inside this month’s *Cytopathology*: The aims of this study are to assess whether the amount of time a cytology learner spent reviewing slides correlates with increased diagnostic competency, and to determine whether time spent reviewing slides immediately after the multi‐head sessions correlates with a higher level of proficiency. The paper also seeks to explore the impacts of the time of day at which slides were reviewed on diagnostic learning outcome.

## Graphical abstract

Boxplot of Bottom of Class for the Exam vs Time Spent in Glass Slide Library.

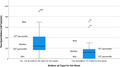

## 1 INTRODUCTION

How best to educate and train cytologists is a challenge faced by many of the universities' training programs and hospitals across the globe. Providing a competent cytology diagnostician with good morphology skills is a vital role that educators play to ensure that the future of the pathology workforce is strong and able to meet the future demands of the profession.^1^ Currently, many cytology screeners are able to independently sign‐out negative gynaecological cases, as well as provide pre‐screening and perform rapid onsite evaluation (ROSE) for specimen adequacy.^2^, ^3^ In the future, the cytology practice for the technologists may involve greater non‐traditional technical responsibilities, including correlation, review of discrepant cases, teaching pathology residents, pre‐screening non‐gynaecological and biopsy cases, and perhaps even giving a preliminary diagnosis as a billable procedure.^2^, ^3^, ^4^, ^5^, ^6^ All of these current and future roles entail a cytology workforce with a strong foundation of morphology skills. The medical and research literature have produced mixed results when it comes to establishing the key aspects of educating a competent workforce.

The traditional perspective is that the path to success and attaining competency lies in exposure to the material, including reviewing what was taught in class within a 24‐hour time frame to reinforce the memory curve for what was learned.^7^, ^8^, ^9^, ^10^ While the traditional view may seem reasonable, Nonis and Hudson studied business students and found that, contrary to popular belief, the amount of time spent studying had no direct influence on developing the learners' competencies.^11^ In fact, several research studies drawn from economic education have concluded that spending too much time studying has a negative effect on the learner’s performance.^12^, ^13^, ^14^

This paper seeks to assess whether the traditional perspective, that perseverance will reap rewards, still holds in cytology education by reviewing the laboratory logs and finding out if there is a correlation between the number of hours spent in the glass slide library looking through the microscope and achieving diagnostic competency, as measured by slide test scores. The use of unknown case slide exams as proxy markers for cytomorphology competency is widespread in cytopathology training and can serve as a good indicator for diagnostic competency.^15^

### 1.1 Chronobiology and studying habits

Chronobiology is the study of biological rhythms and its influence on performance, and this subject has been gaining attention in recent years in healthcare practices, from when to perform a procedure like a colonoscopy, to how time of day influenced the abnormal detection rate of cytology screeners in laboratories.^16^, ^17^ Singh and colleagues conducted a retrospective review of 2305 colonoscopies performed by 18 gastroenterologists over a span of 4 years and found that there is a significant difference in the rate of detection of adenoma between procedures performed in the morning compared to those performed in the afternoon.^18^ Another study of 2087 procedures yielded similar results, where colonoscopies performed in the morning have a lower failure rate than those scheduled in the afternoons.^19^ Researchers at Duke University who analysed over 90,000 surgeries have also found a similar pattern, where surgeries that began in the morning appeared to be less likely to have complications than those that began in the afternoon.^20^ While there is a paucity of chronobiology studies in pathology, there is a study from a cytopathology laboratory that suggests there is a significant difference between the abnormal detection rates of cytology screeners when reviewing cases in the morning vs the afternoon.^21^ Furthermore, the study observed that cytology screeners' abnormal rates declined as the day wore on, further support the idea that more studies should be performed to explore how time influences performance in a variety of settings.^21^

We suspect, given the better detection rates from the various diagnostic procedures and screening tests, that students who spent time in the morning reviewing slides at the laboratory should also achieve better competency in the form of higher slide test scores. Despite past studies on the topic, there is little research focusing on the training of cytologists and looking at this from the perspective of chronobiology. The aim of this study is threefold: (1) To assess whether the amount of time spent reviewing slides correlates with increased diagnostic competency in the form of higher slide test scores; (2) to determine whether time spent reviewing slides within 24‐hours of the multi‐head class session correlates with higher competency; and (3) to explore the impacts of the time of day at which slides were reviewed, if any, on learner's proficiency in the diagnostic skills.

## 2 MATERIALS AND METHODS

The student data were obtained through the student laboratory screening logs that were kept as part of the cytopathology laboratory's education quality assurance and control protocol (IRB Study ID# 2021002516). The study sample included all first‐year cytology students (*n* = 9) and occurred between September and November 2021. As the first‐year students came into the cytology lab to access the facility's glass slide library, they recorded the date, the time at which they entered the lab, the time at which they exited the lab, the number of slides/cases reviewed, and any issues they may have experienced into the facility log binder. The completed logs for the months of September through November, representing all the unit slide tests for the class, were accessed to reconstruct the relationship between the number of hours the students spent reviewing glass slides on their microscope and the corresponding slide test result for the weekly units. A total of seven slide competency tests were given during the semester, corresponding to the seven major units in the gynaecological diagnostic cytology course, ranging from the morphology of benign cellular changes and infectious agents to malignant squamous and glandular lesions and their corresponding precursors. To assess the relationship between time spent and test score (objective 1), each student's weekly results for the seven slide tests and the number of hours spent in the laboratory reviewing morphology were plotted and then analysed using the non‐parametric Spearman rank correlations. A similar analysis was also performed comparing the number of slides reviewed against test scores. According to the Shapiro–Wilk test of normality and a visual inspection of the histogram, time spent is not normally distributed (*P* = 0.001). Moreover, since the sample is small with a few outliers, the non‐parametric Spearman rank correlation was used instead of the Pearson correlation. All self‐reported times calculated from the logs were isolated for each unit review only and were not cumulative.

Additionally, the students' diagnostic competency scores were classified each week into two categories, highest third and lowest third, defined as the highest one‐third of the class and lowest one‐third of the class for the week, respectively.^22^ Bottom‐of‐class scores were equal to or less than 55% for unit 1, 67% for unit 2, 28% for unit 3, 62% for unit 4, 50% for unit 5, 63% for unit 6, and 72% for unit 7. Similarly, top‐of‐class scores were equal to or greater than 89% for unit 1, 89% for unit 2, 56% for unit 3, 78% for unit 4, 72% for unit 5, 72% for unit 6, and 83% for unit 7. For each of the 63 test scores (nine students over seven tests), a ‘1’ was assigned if the test score was in the bottom third of the class that week, and a ‘0’ was given for the rest. A point‐biserial Pearson correlation was used to assess if whether there was a relationship between time spent and scoring at the bottom of the class.

To evaluate the influence of specific slide reviewing habits, specifically whether studying slides within a 24‐hour period following a multi‐head session is related to improved morphological competency (objective 2), the Spearman correlation was performed, comparing time spent reviewing within the 24‐hour period and test scores. According to the Shapiro–Wilk test of normality and a visual inspection of the histogram, time spent studying within 24 hours (recency) of the session is not normally distributed (*P* < 0.001).

Finally, to assess whether the time of day at which slides are reviewed affects test results and competency (objective 3), data was split into morning and afternoon, using 12 noon as the cutoff, where ‘0’ was assigned for am and ‘1’ was given for pm. A contingency table for time of day (am/pm) with bottom‐of‐class (yes/no) was created, and a chi‐square test was subsequently performed to appraise the relationships between the variables. All statistical analyses were performed using SPSS version 28 (IBM), and significance was assumed at *P* < 0.05.

## 3 RESULTS

**FIGURE 1:**
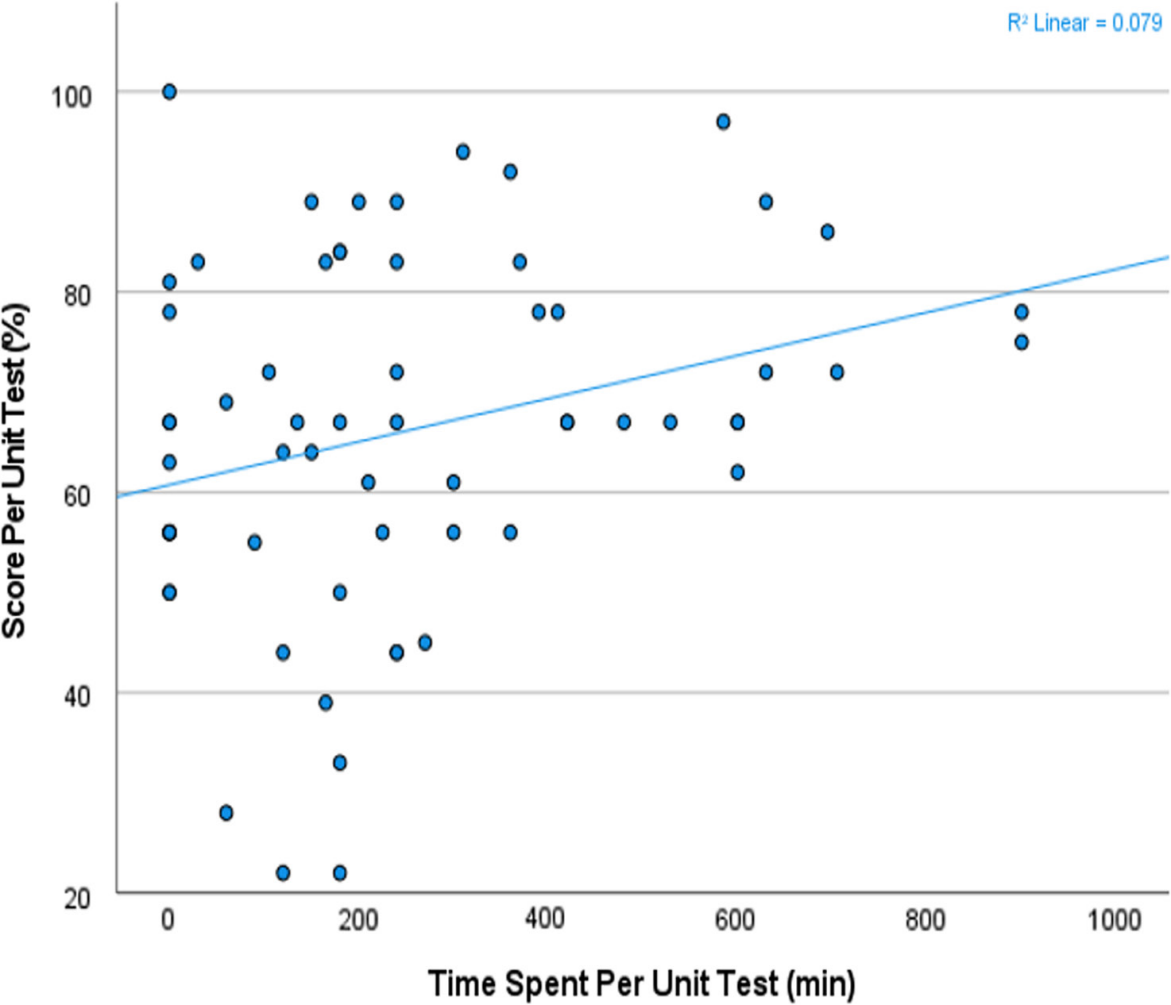
Scatter plot of unit score by time spent

From the 63 test results, there was a significant positive linear relationship (*R* = 0.292, *P* = 0.020) between the number of hours students spent in the microscopy laboratory reviewing cases and their competency, represented by their test scores (Figure 1). Those who spent less time reviewing slides had lower competency scores; conversely, those who spent more time reviewing slides had higher competency scores. However, there are several outliers at the lower extreme, specifically the three that scored above 80% on the slide tests, represented by three different students on different unit tests, who spent minimal time at the laboratory reviewing slides. Interestingly, when test score was analysed from the perspective of the number of slides reviewed, no correlation was found (Table 1).

**TABLE 1:** Summary of objectives, variables, and test results

	Objectives	Variables	Significance
1	To assess whether the amount of time spent reviewing slides correlates with increased diagnostic competency	Time spent for each unit test (min) vs test scores for each unit test (%)	Spearman: *R* = 0.292, *P* = 0.020
		Time spent for each unit test (min) vs scoring at the bottom‐third of the class or not (0 = not in bottom‐third of class; 1 = bottom‐third of class)	Point‐biserial: *R* = −0.331, *P* = 0.008
		Number of slides reviewed vs unit test score (%)	Spearman: *R* = 0.155, *P* = 0.226
2	To determine whether time spent reviewing slides within 24 hours of the multi‐head class session correlates with higher competency	Time spent reviewing (min) within 24 h of multi‐head vs test scores for each unit test	Spearman: *R* = 0.044, *P* = 0.731
3	To explore the impacts of the time of day at which slides were reviewed on slide competency (slide test scores)	Time of day (am or pm) of reviewing slides vs scoring at the bottom‐third of the class or not (0 = not in bottom‐third of class; 1 = bottom‐third of class)	*χ* ^2^ = 1.62, *P* = 0.41

**FIGURE 2:**
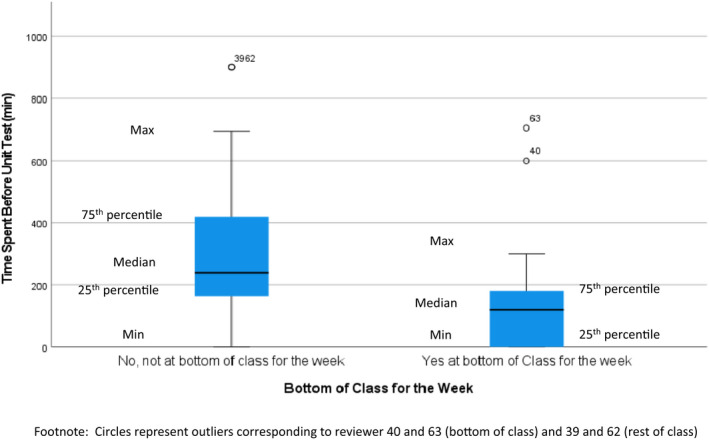
Boxplot of bottom‐of‐class scores for the exam vs time spent in the glass slide library. Circles represent outliers corresponding to reviewer 40 and 63 (bottom of the class) and 39 and 62 (rest of the class)

When the cytology students' unit diagnostic test scores were classified into low and high categories (Table 1), there was a significant correlation (R = −0.33, P = 0.008) between a lack of time spent screening slides in the lab and the number of times a student was ranked at the bottom of the class in the unit tests (Figure 2).

**FIGURE 3:**
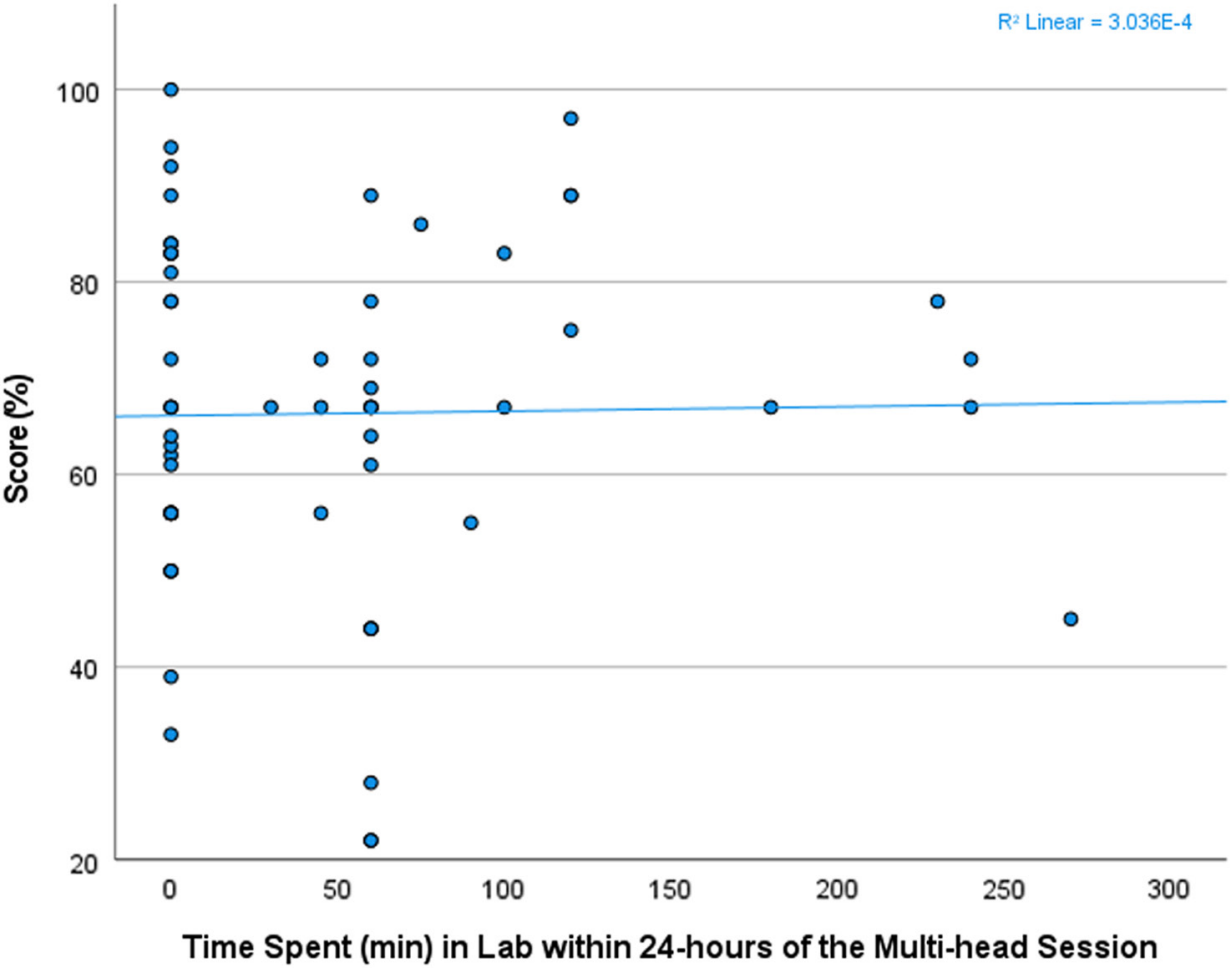
Scatterplot of time spent in the microscopy lab within 24 hours of a multi‐head lesson vs test score

In Figure 3, a scatterplot showing the time the students spent in the microscopy lab within the 24‐hour period following the cytology class lesson and their diagnostic competency scores showed mixed results, with instances (*n* = 7) of students achieving test scores greater than 80% who did not spend any time reviewing slides within the 24‐hour period following the lesson at the lab. There is no relationship between immediate reinforcement, as defined by a review within 24 hours, and test scores (*R* = 0.044, *P* = 0.731). It is interesting to note that 7 students scored over 80% with no reinforcement, with another 5 scoring over 80% with reinforcement.

**FIGURE 4:**
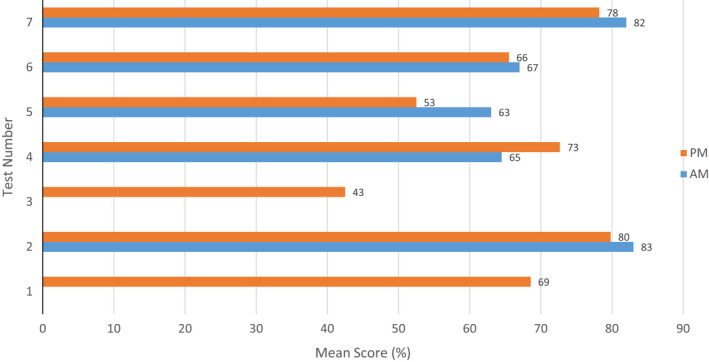
Mean score by test number and time of day

Finally, the data do not support the difference in test scores between those who reviewed cases in the morning vs those in the afternoon (χ^2^[*df* = 1, *n* = 46] = 1.623, *P* > 0.05). In looking at the weekly average scores for those who studied in the morning vs the afternoon, the morning cohort appeared to be just a few percentage points higher in test numbers 2, 5, 6, and 7 but lower in number 4 (Figure 4). It is also worth noting that in the unit 7 test, which had one of the higher mean scores for the semester, the two top scorers spent a total of 530 min while the bottom two students did not spend time at all in the morning at the microscopy lab.

## 4 DISCUSSION

This article addresses the critical issue of how best to educate and train the next generation of cytologists, and how to provide them with a strong foundation in cytomorphology that enables them to face the professional challenges of tomorrow. As expected, there is a significant correlation between those who are willing to roll up their sleeves and put in the hours in front of the microscope and improved diagnostic competency. Surprisingly, the time spent reviewing cases in the period immediately after multi‐head sessions and the time of day at which slides were reviewed were not significantly correlated with increased cytomorphology competency.

As expected, the number of hours a cytology learner spent reviewing slides correlates with slide competency, as demonstrated by the routine slide tests taken throughout the course. While the idea that the path to proficiency in a subject lies in repeated interactions may not necessarily be the case in theoretically heavy subjects such as economics or business, we hypothesize that a more hands‐on discipline benefits from repeated interactions. Many pathology residents have recognised the need to spend more time studying to obtain expertise in cytopathology, and the importance of such an endeavour, and have opted to undertake an additional year‐long fellowship in the field to gain mastery of cytomorphology skills.^15^ Our cytology student log review study results confirmed the connection between time spent reviewing cytology study sets and improved cytomorphology competence. Moreover, our observations showed that there is a correlation between bottom‐of‐class test scores and not spending time reviewing the study sets. In other words, time spent in the lab has a protective effect against scoring low on the exam, and this finding that should provide reassurance to those who may not be fast learners that if they put in the effort, they will be able to develop adequate cytomorphological competence.

An interesting observation was that, contrary to our expectations, neither reviewing slides within a 24‐hour period following the multi‐head session lecture nor the time of day at which slides were reviewed were correlated with diagnostic competency in our cytology student population. It may be that there is no connection between chronobiology and optimal studying habits, or that our study population does not follow the chronobiology of the general population. People do not all experience a day in the same way, and each of us has an internal clock governing our optimal wake–sleep patterns, known as a ‘chronotype’. College and graduate students' chronotype or wake–sleep pattern is an anomaly that differs from that of the general population, which can help to explain why the traditional morning and afternoon study time distinction we hypothesized would be observed may not be appropriate for this population.^16^, ^23^ Regarding the recency effect, it appeared, based on a review of the trend (Figure 3), that there is a point of diminishing returns, such that those who spent more than 230 min reviewing cases immediately after the multi‐head session will not achieve additional benefits beyond that 230 min of study.

The finding that it is the amount of time spent reviewing cases, not the actual number of slides viewed, that was correlated with unit score is intriguing. While it seemed intuitive that the more cases the students were able to review, the better prepared the learners will be, others have suggested that it is not the quantity per se but the deliberate engagement with the new material that leads to competency and proficiency in medical‐related skillsets.^24^ Perhaps the question is not how many slides a student had reviewed or how long someone had spent at the lab, but the quality of that engagement. For a cytologist developing both locator and interpretive skills, it may take time to achieve the proper hand‐eye coordination that is needed to screen and locate the infectious organisms or the dysplastic cells. Both skill‐sets require continued self‐monitoring and feedback from instructors to improve over time. Future studies on how best to build morphology skills would benefit from exploring broader constructs such as motivational attitudes and self‐efficacy in the context of how such factors can improve screening habits to enhance learning.

This study has several limitations. First, the results are based on students self‐reporting and are prone to error, either inadvertently or deliberately. Second, the retrospective review is focused on gynaecological cytology and may not be generalizable to all types of courses. Third, the emphasis of the study is on the relationship between time spent, study habits, and subsequent competency at the unit test score level, so little demographic information was obtained. Fourth, the small size of the sample (*n* = 9) may have impacted the power of the study to draw conclusions. Finally, the time period over which the study was conducted, one 3‐month period between September and November, may have introduced inadvertent seasonality biases as well.

## 5 CONCLUSION

Despite the limitations, we believe our results to be informative and to have important implications for providing insights into how best to implement morphology learning. First, it reiterated the importance of spending time in the laboratory for the development of competency in the subject. Second, it provided hope for the cytology students who may be struggling by demonstrating that if you can allocate time to reviewing study‐sets, you are less likely to end up at the bottom of the class. In sum, educating and training cytologists so that they are equipped with a strong foundation in morphology may be challenging, but with the right message, on the importance of putting in the hours in the microscopy lab, the challenge can be overcome.

## CONFLICT OF INTEREST

The author has no conflict of interest to declare.

## AUTHOR CONTRIBUTIONS

Paul Z. Chiou: Conceptualized and designed the study, evaluated data, interpreted results, and wrote the manuscript. Yuane Jia: Evaluated data, interpreted results, review, and edited the manuscript.

## Data Availability

The data that support the findings of this study are available from the corresponding author, PZC, upon reasonable request.

## References

[1] Chiou PZ , Mulder L , Jia Y . On pathology laboratory recruitment and retention: insights from the 16 personalities type indicators. Am J Clin Pathol. 2021;156(4):625‐633. 33728424 10.1093/ajcp/aqaa257

[2] Anic V , Eide M . Survey of training and education of cytotechnologists in Europe. Cytopathology. 2014;25(5):302‐306. 25099770 10.1111/cyt.12168

[3] Donnelly A . The ‘Morph’ology of cytotechnology education. Cytopathology. 2016;27(5):310‐312. 27650597 10.1111/cyt.12371

[4] Chiou PZ . Employer expectations for the MS‐level cytology practitioner: a regional perspective. Am J Clin Pathol. 2020;153(4):487‐496. 31833534 10.1093/ajcp/aqz185

[5] Cohen MB , Perez‐Reyes N , Stoloff AC . The status of residency training in cytopathology. Diagn Cytopathol. 1995;12(2):186‐187. 7774504 10.1002/dc.2840120222

[6] Wilson A . The role of Cytotechnologists in quality assurance and audit in non‐gynaecological cytology. Cytopathology. 2015;26(2):75‐78. 25800805 10.1111/cyt.12246

[7] Campus wellness . Curve of Forgetting. University of Waterloo; 2021. https://uwaterloo.ca/campus‐wellness/curve‐forgetting. Accessed December 29, 2021.

[8] Michaels JW , Miethe TD . Academic effort and college grades. Soc Forces. 1989;68(1):309‐319.

[9] Shain DD . Study Skills and Test‐Taking Strategies for Medical Students: Find and Use Your Personal Learning Style. Springer Science & Business Media; 2012.

[10] Wolf FM , Ulman JG , Saltzman GA , Savickas ML . Allocation of time and perceived coping behavior of first‐year medical students. Acad Med. 1980;55(11):956‐958. 10.1097/00001888-198011000-00011 7441679

[11] Nonis SA , Hudson GI . Academic performance of college students: influence of time spent studying and working. J Educ Bus. 2006;81(3):151‐159.

[12] Becker WE . Teaching economics to undergraduates. J Econ Lit. 1997;35(3):1347‐1373.

[13] Krohn GA , O’Connor CM . Student effort and performance over the semester. J Econ Educ. 2005;36(1):3‐28.

[14] Siegfried JJ , Fels R . Research on teaching college economics: a survey. J Econ Lit. 1979;17(3):923‐969.

[15] Yu GH . Goals and guidelines for residency training in cytopathology. Diagn Cytopathol. 2011;39(6):455‐460. 20730903 10.1002/dc.21436

[16] Pink DH . When: The Scientific Secrets of Perfect Timing. Penguin Press; 2019.

[17] Smolensky MH . Chronobiology and chronotherapeutics applications to cardiovascular medicine. Am J Hypertens. 1996;9(4):11S‐21S. 8722412 10.1016/0895-7061(95)00405-x

[18] Singh S , Dhawan M , Chowdhry M , Babich M , Aoun E . Differences between morning and afternoon colonoscopies for adenoma detection in female and male patients. Ann Gastroenterol. 2016;29(4):497. 27708517 10.20524/aog.2016.0079 PMC5049558

[19] Sanaka MR , Shah N , Mullen KD , Ferguson D , Thomas C , McCullough AJ . Afternoon colonoscopies have higher failure rates than morning colonoscopies. Am J Gastroenterol. 2006;101(12):2726‐2730. 17227519 10.1111/j.1572-0241.2006.00887.x

[20] Wright MC , Phillips‐Bute B , Mark J , et al. Time of day effects on the incidence of anesthetic adverse events. Qual Saf Health Care. 2006;15(4):258‐263. 16885250 10.1136/qshc.2005.017566 PMC2564010

[21] Elsheikh TM , Kirkpatrick JL , Fischer D , Herbert KD , Renshaw AA . Does the time of day or weekday affect screening accuracy? A pilot correlation study with cytotechnologist workload and abnormal rate detection using the ThinPrep Imaging System. Cancer Cytopathol. 2010;118(1):41‐46. 20099317 10.1002/cncy.20060

[22] Braham L . Crowds know best, says Morningstar. But do they? In. Barron’s. Dow Jones & Company; 2021:1. Vol CI.

[23] Roenneberg T , Kuehnle T , Pramstaller PP , et al. A marker for the end of adolescence. Curr Biol. 2004;14(24):R1038‐R1039. 15620633 10.1016/j.cub.2004.11.039

[24] Ericsson KA . Deliberate practice and the acquisition and maintenance of expert performance in medicine and related domains. Acad Med. 2004;79(10):S70‐S81. 10.1097/00001888-200410001-00022 15383395

